# Effects of Sphingomyelin-Containing Milk Phospholipids on Skin Hydration in UVB-Exposed Hairless Mice

**DOI:** 10.3390/molecules27082545

**Published:** 2022-04-14

**Authors:** Yejin Ahn, Min Guk Kim, Kyungae Jo, Ki-Bae Hong, Hyung Joo Suh

**Affiliations:** 1 Department of Integrated Biomedical and Life Science, Graduate School, Korea University, Seoul 02841, Korea; ahnyj708@gmail.com (Y.A.); minguk94@gmail.com (M.G.K.); kyungae11@korea.ac.kr (K.J.); 2 Department of Food Science and Nutrition, Jeju National University, Jeju 63243, Korea; 3 BK21FOUR R&E Center for Learning Health Systems, Korea University, Seoul 02841, Korea

**Keywords:** milk phospholipids, sphingomyelin, skin hydration, Nrf2, hyaluronic acid

## Abstract

Reactive oxygen species (ROS) generated by ultraviolet (UV) exposure cause skin barrier dysfunction, which leads to dry skin. In this study, the skin moisturizing effect of sphingomyelin-containing milk phospholipids in UV-induced hairless mice was evaluated. Hairless mice were irradiated with UVB for eight weeks, and milk phospholipids (50, 100, and 150 mg/kg) were administered daily. Milk phospholipids suppressed UV-induced increase in erythema and skin thickness, decreased transepidermal water loss, and increased skin moisture. Milk phospholipids increased the expression of filaggrin, involucrin, and aquaporin3 (AQP3), which are skin moisture-related factors. Additionally, hyaluronic acid (HA) content in the skin tissue was maintained by regulating the expression of HA synthesis- and degradation-related enzymes. Milk phospholipids alleviated UV-induced decrease in the expression of the antioxidant enzymes superoxidase dismutase1 and 2, catalase, and glutathione peroxidase1. Moreover, ROS levels were reduced by regulating heme oxygenase-1 (HO-1), an ROS regulator, through milk phospholipid-mediated activation of nuclear factor erythroid-2-related factor 2 (Nrf2). Collectively, sphingomyelin-containing milk phospholipids contributed to moisturizing the skin by maintaining HA content and reducing ROS levels in UVB-irradiated hairless mice, thereby, minimizing damage to the skin barrier caused by photoaging.

## 1. Introduction

The skin is the primary protective barrier of the human body, and it plays a role in preserving moisture in the body and protecting the skin from the external environment. The epidermal stratum corneum, the outermost layer of the skin, is involved in protecting the moisture content of the skin in a dry environment [1]. When the epidermis is repeatedly exposed to a large amount of ultraviolet (UV) light, it induces reactive oxygen species (ROS) generation and oxidative stress. UV rays can penetrate the epidermal and dermal layers and facilitate ROS generation in cells and tissues through various processes [2]. UV-induced ROS accelerate aging by inducing photooxidative damage to the skin. ROS cause an imbalance in enzymatic and non-enzymatic antioxidant defense systems of the skin and prevent normal cell functions owing to lipid peroxidation-induced cell membrane damage [3]. Increased oxidative stress in skin cells activates the expression of matrix metalloproteases, thereby reducing collagen production and elastic fiber synthesis, thereby promoting skin aging [4]. As it cannot recover from the continuous oxidation state, the skin surface becomes rough and loses its luster, leading to skin aging, which involves loss of elasticity and wrinkle formation [5].

The stratum corneum, which affects skin moisture retention, forms a lipid layer composed of ceramide, cholesterol, and free fatty acids between keratinocytes and has low permeability compared to general phospholipid biofilms, thereby inhibiting the permeation of external substances [6]. The stratum corneum produces natural moisturizing factors, such as amino acids, lactic acid, urea, citrate, and hyaluronic acid (HA), to maintain body water balance [7]. The stratum corneum of a healthy person contains approximately 10 to 30% moisture, and insufficient moisture causes abnormalities in the skin barrier and increases transdermal moisture loss, resulting in dry skin. Loss of moisture caused by abnormal skin barrier function reduces skin elasticity, thickens the epidermis, promotes wrinkle formation, causes diseases such as itching and xerosis, and worsens diseases such as psoriasis and atopic dermatitis [8]. Moisture supply and maintenance of moisture in the skin are important in terms of pathological and cosmetic aspects [7]. Active ingredients required to moisturize the skin include ceramide, hydroxy acid, glycerin, and butylene glycol, and these compounds are either applied to the skin or ingested orally [9,10].

Research on the development of commercial cosmeceuticals has been conducted through the repositioning of natural and synthetic products, and research to explore and utilize food materials with wrinkle-improving effects continues steadily [11]. This study used sphingomyelin-containing phospholipids, which are polar lipids extracted from milk whey with ethanol. Most of it contains phospholipids, mainly phosphatidylcholine and phosphatidylethanolamine, and sphingolipids, mainly sphingomyelin [12]; therefore, it is a food ingredient rich in precursors of ceramides necessary for skin moisturizing. Milk fat is a dietary source of sphingomyelin, and dietary sphingomyelin raises ceramide levels in the body. Ceramide plays a role in maintaining the moisture in the epidermis and skin barrier function [13,14].

Numerous studies have been conducted on natural diets and herbs for skin moisturizing and skin barrier function improvement, but studies on milk phospholipids are limited. Therefore, in this study, the skin moisturizing effect of milk phospholipids was evaluated by measuring the expression levels of skin hydration factors and enzymes related to the synthesis and decomposition of hyaluronic acids in hairless mice induced by photoaging. By measuring the expression level of enzymes involved in ROS removal by milk phospholipids, the potential for skin photoaging inhibition was evaluated, and the mechanism of action of ROS removal was investigated.

## 2. Results

### 2.1. Effects of Milk Phospholipids on Body Weight Changes and Plasma Biochemical Parameter

During the experimental period, all the experimental groups showed a tendency to gradually increase in body weight (Supplementary Materials Table S1). In addition, the milk phospholipid administration groups (low-dose milk phospholipids [ML]: 50 mg/kg; medium-dose milk phospholipids [MM]: 100 mg/kg; high-dose milk phospholipids [MH]: 150 mg/kg) did not show a significant difference in body weight compared to the NOR group. Plasma biochemical values are shown in Table S2. Plasma levels of glucose, aspartate transaminase and alanine transferase were not significantly different between groups. The milk phospholipid administration groups (MM and MH) showed significantly lower triglyceride levels compared to the NOR group (*p* < 0.05 and *p* < 0.01, respectively), but the total cholesterol levels were similar to the NOR group.

### 2.2. Effects of Milk Phospholipids on Skin Parameters

**Figure 1:**
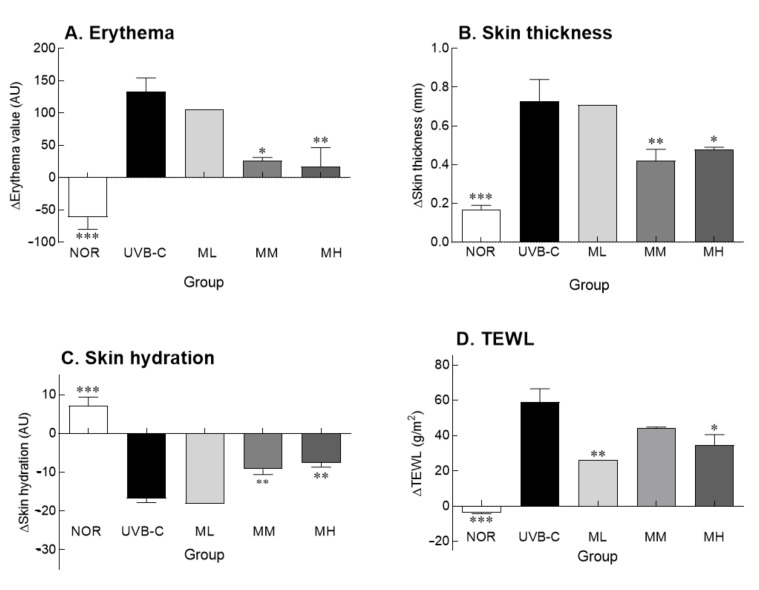
Effects of milk phospholipids on erythema formation (**A**), skin thickness (**B**), skin hydration (**C**) and transepidermal water loss (TEWL) (**D**) in ultraviolet (UV) B-irradiated hairless mice. NOR: oral administration of saline without UVB irradiation; UVB-C: oral administration of saline under UVB irradiation; ML: oral administration of low-dose (50 mg/kg b.w.) milk phospholipids under UVB irradiation; MM: oral administration of medium-dose (100 mg/kg b.w.) milk phospholipids under UVB irradiation; MH: oral administration of high-dose (150 mg/kg b.w.) milk phospholipids under UVB irradiation. Data are expressed as means ± standard error (*n* = 6). * *p* < 0.05, ** *p* < 0.01, and *** *p* < 0.001 vs. UVB-C group (Tukey’s test).

Erythema formation and skin thickness are expressed as delta values, which represent the difference in values before and after the UV treatment (Figure 1A,B). As representative phenomena of skin photoaging, the erythema index and skin thickness were significantly higher in the UVB-C group than in the normal group (*p* < 0.001). However, oral administration of milk phospholipids decreased the erythema index in a concentration-dependent manner, showing improvement in UV-induced photoaging (Figure 1A). Administration of medium (MM) and high (MH) doses of milk phospholipids significantly decreased the erythema index compared to the UVB-C group (*p* < 0.05 and *p* < 0.01, respectively). Oral milk phospholipid administration, particularly MM and MH doses, significantly decreased UV-mediated increase in skin thickness compared to the UVB-C group (Figure 1B; *p* < 0.01 and *p* < 0.05, respectively). Collectively, MM and MH groups showed positive effects on erythema and skin thickness.

Skin hydration and transepidermal water loss (TEWL) were determined to evaluate skin barrier function, which plays an important role in skin hydration. Skin moisture content and transdermal moisture loss are expressed as delta values, which represent the difference in values before and after the experiment (Figure 1C,D). There were significant differences in the delta values of skin hydration and TEWL between normal and UVB-C groups (*p* < 0.001). Furthermore, UV-induced reduction in skin hydration was reversed by milk phospholipid administration in a concentration-dependent manner (Figure 1C). Similarly, milk phospholipid administration also improved TEWL (Figure 1D). In particular, ML and MH groups showed a significant improvement in TEWL compared to the UVB-C group, but there was no dose-dependent change (*p* < 0.01 and *p* < 0.05, respectively). Compared with the UVB-C group, the MM group showed a tendency to decrease TEWL, but there was no significant difference. Taken together, milk phospholipids exhibited improving effects on the skin barrier function.

### 2.3. Effects of Milk Phospholipids on HA Synthesis and Degradation

**Figure 2:**
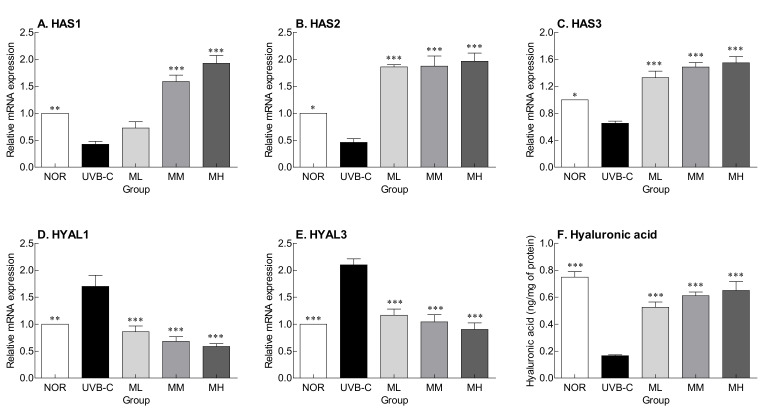
Effects of milk phospholipids on gene expression of HAS (**A**–**C**) and HYAL (**D**,**E**) and hyaluronic acid (HA) content (**F**) in UVB-irradiated hairless mice. NOR: oral administration of saline without UVB irradiation; UVB-C: oral administration of saline under UVB irradiation; ML: oral administration of low-dose (50 mg/kg b.w.) milk phospholipids under UVB irradiation; MM: oral administration of medium-dose (100 mg/kg b.w.) milk phospholipids under UVB irradiation; MH: oral administration of high-dose (150 mg/kg b.w.) milk phospholipids under UVB irradiation. Data are expressed as means ± standard error (*n* = 6). * *p* < 0.05, ** *p* < 0.01, and *** *p* < 0.001 vs. UVB-C group (Tukey’s test). HAS: hyaluronan synthase; HYAL: hyaluronidase.

HA is a compound responsible for skin moisture and is involved in inhibiting moisture loss from the epidermis and maintaining skin elasticity. HA is synthesized by hyaluronic acid synthase (HAS) and degraded by hyaluronidase (HYAL). UV irradiation significantly decreased the expression of HAS (HAS1, 2, and 3) (Figure 2A–C; *p* < 0.01, *p* < 0.05, *p* < 0.05, respectively), but significantly increased the expression of HYAL (HYAL1 and 3) (Figure 2D,E; *p* < 0.01, *p* < 0.001, respectively) compared to the normal group. However, milk phospholipids increased HAS expression, which were reduced by UV, and decreased HYAL expression, which were increased by UV, in a concentration-dependent manner (Figure 2D,E). HA content in the skin was also reduced by UV irradiation, but it was significantly increased by oral milk phospholipid administration (Figure 2F; *p* < 0.001). Hereby, milk phospholipids are thought to improve skin hydration by regulating the expression of HAS and HYAL.

### 2.4. Effects of Milk Phospholipids on the Expression of Skin Moisture-Related Factors

**Figure 3:**
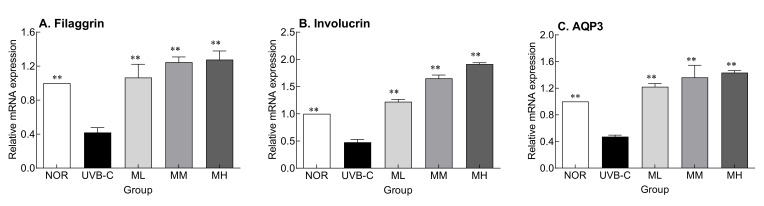
Effects of milk phospholipids on the expression of filaggrin (**A**), involucrin (**B**), and AQP3 (**C**) in UVB-irradiated hairless mice. NOR: oral administration of saline without UVB irradiation; UVB-C: oral administration of saline under UVB irradiation; ML: oral administration of low-dose (50 mg/kg b.w.) milk phospholipids under UVB irradiation; MM: oral administration of medium-dose (100 mg/kg b.w.) milk phospholipids under UVB irradiation; MH: oral administration of high-dose (150 mg/kg b.w.) milk phospholipids under UVB irradiation. Data are expressed as means ± standard error (*n* = 6). ** *p* < 0.01 vs. UVB-C group (Tukey’s test). AQP3: aquaporin3.

UV rays damage the skin, causing abnormal skin barrier function and eventually dryness [15]. The effect of milk phospholipids on the recovery of skin barrier function damaged by UV was examined. The gene expression of involucrin and filaggrin, which are differentiation-promoting factors involved in keratinocyte membrane formation, and AQP3, a gene that encodes a protein that synthesizes the water passage in the basal outer layer of the cell membrane, were determined (Figure 3). Their expressions were significantly lower in the UVB-C group than in the normal group (Figure 3; *p* < 0.01, *p* < 0.001, and *p* < 0.01, respectively). Milk phospholipids significantly increased their expression in a concentration-dependent manner (Figure 3; *p* < 0.001). Collectively, milk phospholipids appear to be involved in the restoration of skin barrier function by suppressing UV-mediated decrease in the expression of these factors.

### 2.5. Effects of Milk Phospholipids on ROS Production and Expression of Genes Encoding Antioxidant Enzymes

**Figure 4:**
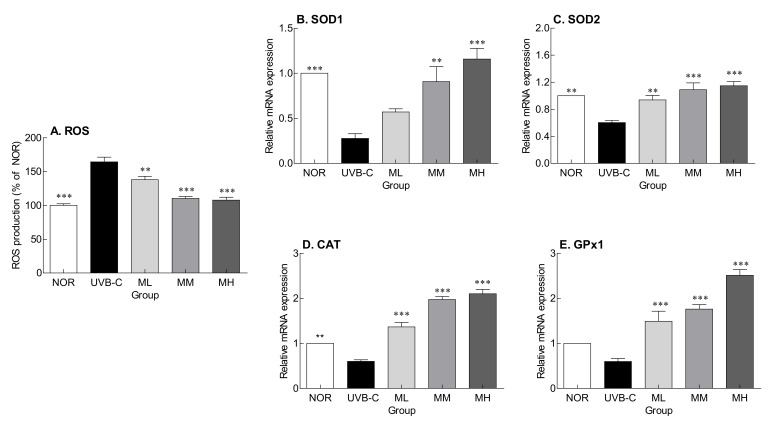
Effects of milk phospholipids on ROS production (**A**) and the expression of genes encoding the antioxidant enzymes SOD1 (**B**), SOD2 (**C**), CAT (**D**), and GPx1 (**E**) in UVB-irradiated hairless mice. NOR: oral administration of saline without UVB irradiation; UVB-C: oral administration of saline under UVB irradiation; ML: oral administration of low-dose (50 mg/kg b.w.) milk phospholipids under UVB irradiation; MM: oral administration of medium-dose (100 mg/kg b.w.) milk phospholipids under UVB irradiation; MH: oral administration of high-dose (150 mg/kg b.w.) milk phospholipids under UVB irradiation. Data are expressed as means ± standard error (*n* = 6). ** *p* < 0.01 and *** *p* < 0.001 vs. UVB-C group (Tukey’s test). ROS: reactive oxygen species; CAT: catalase; SOD: superoxide dismutase; Gpx-1: glutathione peroxidase-1.

Figure 4 shows the inhibitory effect of milk phospholipids on ROS production and expression of genes encoding antioxidant enzymes. ROS levels were significantly higher in the UVB-C group than in the normal group (Figure 4; *p* < 0.001). Milk phospholipids significantly lowered UV-induced ROS production in a concentration-dependent manner (Figure 4A; *p* < 0.01 and *p* < 0.001, respectively). The expression of superoxide dismutase 1 (SOD1), SOD2, catalase (CAT), and glutathione peroxidase 1 (GPx1), which are involved in ROS removal, were lower in the UVB-C group than in the normal group (Figure 4B–E). Milk phospholipids suppressed UV-mediated decrease in gene expression in a concentration-dependent manner. In particular, the expression of the antioxidant enzymes was significantly increased by MM and MH doses. Altogether, oral milk phospholipid administration inhibited photoaging by suppressing ROS generation and regulating the expression of genes encoding antioxidant enzymes.

### 2.6. Effects of Milk Phospholipids on Nrf2-Keap1-Related Protein Expression

**Figure 5:**
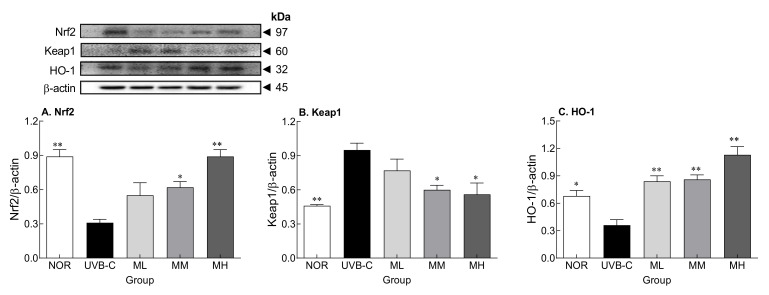
Effects of milk phospholipids on protein expression of Nrf2 (**A**), Keap1 (**B**), and HO-1 (**C**) in UVB-irradiated hairless mice. Western blot and protein quantifications are shown. NOR: oral administration of saline without UVB irradiation; UVB-C: oral administration of saline under UVB irradiation; ML: oral administration of low-dose (50 mg/kg b.w.) milk phospholipids under UVB irradiation; MM: oral administration of medium-dose (100 mg/kg b.w.) milk phospholipids under UVB irradiation; MH: oral administration of high-dose (150 mg/kg b.w.) milk phospholipids under UVB irradiation. Data are expressed as means ± standard error (*n* = 6). * *p* < 0.05 and ** *p* < 0.01 vs. UVB-C group (Tukey’s test). Nrf2: Nuclear factor erythroid-2-related factor 2; Keap1: Kelch-like ECH-associated protein 1; HO-1: heme oxygenase-1.

Oral administration of milk phospholipids suppressed UVB-induced ROS generation and the decrease in gene expression of antioxidant enzymes. Therefore, to examine the underlying mechanisms of milk phospholipids, protein expression of nuclear factor erythoride-2-related factor 2 (Nrf2) and Kelch-like ECH-associated protein 1 (Keap-1), which are affected by oxidative stress, and heme oxygenase-1 (HO-1), an antioxidant enzyme, were examined by Western blotting (Figure 5). Protein expression of Nrf2 and HO-1 (*p* < 0.001 and *p* < 0.05, respectively) were significantly lower, but that of keap1, a negative regulator of Nrf2, was significantly higher (*p* < 0.01) in the UVB-C group than in the normal group (Figure 5A,B). Milk phospholipids increased Nrf2 and HO-1 expression and decreased Keap1 expression in a concentration-dependent manner. Taken together, milk phospholipids demonstrated ROS scavenging effects by increasing the expression of the transcription factor Nrf2, contributing to an increase in ROS scavenging-related enzymes.

## 3. Discussion

The skin has a barrier function to protect the body from environmental factors, such as chemicals, pathogens, air pollutants, and UV rays, and a moisturizing function to prevent water loss from the body. The skin is an organ that retains moisture and performs an essential barrier function to protect the body from the intrusion of external factors [16]. In particular, the stratum corneum of the epidermis acts as a skin barrier to protect the skin from the outside while retaining moisture [17]. Skin moisture is the most important factor for maintaining skin health and controlling aging. When UV-induced photoaging progresses, the optimal moisture content of the skin cannot be achieved, and enzymes that produce lipids and natural moisturizing factors are not activated, causing the stratum corneum of the skin to become dry and thick [18].

The keratinocyte membrane is formed when proteins, such as loricrin and involucrin, generated during the differentiation of keratinocytes are cross-linked by transglutaminases [19]. Genes whose expression increases as differentiation progresses include transglutaminase 1 and 3, involucrin, loricrin, cornifin, and filaggrin. Among them, transglutaminase 1 strengthens the skin barrier by crosslinking the structural proteins involucrin, loricrin, and cornifin and imparts resistance and insolubility by catalyzing a stable isotope peptide bond during keratinocyte formation [20]. UV light inhibits the production of natural moisturizing factors by reducing the expression of proteins involved in keratinocyte membrane formation, such as filaggrin, involucrin, and caspase-14 [21]. In keratinocytes, among transmembrane proteins, AQP3 is expressed; aquaporins (AQPs) specifically transport water and glycerol into cells. Glycerol is a structural component for various lipids, and has a positive effect on elasticity and wound healing by increasing the water content of the epidermal layer [22]. Interestingly, AQP3 expression decreases with age, and contributes to skin dryness [23]. As a result, UV-induced damage to the epidermis layer causes skin dryness by reducing skin moisture content [24]. We found that UV rays reduced the expression of genes encoding filaggrin, involucrin, and AQP3, which are skin moisture-related factors; however, oral milk phospholipid administration reversed this effect (Figure 3). Uncontrolled expression of inflammation-related factors causes dysfunction of the epidermal barrier, which is seen in diseases such as atopic dermatitis and psoriasis. Our previous study reported that administration of sphingomyelin-containing milk phospholipids reduces production of proinflammatory cytokines in the photoaged skin [25]. We also confirmed that milk phospholipids had similar effects to virgin coconut oil, *Centella asiatica*, and *Tamnolia vermicularis*, which are known to affect epidermal markers (filaggrin, involucrin and AQP3) responsible for keratinocyte differentiation and skin barrier function [26,27,28].

Although UV irradiation decreased skin moisture and increased TEWL, oral milk phospholipid administration improved the skin barrier damage (Figure 1). UV irradiation promotes the detachment of keratinocytes from the skin surface, weakening the skin hydration and skin barrier function. According to the H&E staining results of epidermis, oral administration of milk phospholipids inhibited the increase in epidermal thickness caused by UV irradiation [29]. In addition, administration of milk phospholipids lowered plasma triglyceride levels compared to the NOR group. Ref. [30] reported that milk-derived phospholipids improved plasma lipid levels, including triglycerides, in obese mice induced by a high-fat diet. UV irradiation induces the increase of TEWL and the alteration of stratum corneum lipid profile by disrupting epidermal barrier functions in skin [31]. According to our previous study [25], sphingomyelin, which is involved in the skin barrier function, decreased when irradiated with UV light, but showed a tendency to increase in the skin tissue by administration of milk phospholipids. Dietary sphingomyelin might improve skin barrier function by altering skin inflammation and covalently bound ω-hydroxy ceramides, and it is also known that dietary sphingomyelin can promote the formation of the epidermal cornified envelope by changes in inflammation-related gene expression [32].

ROS and reactive nitrogenous species (RNS), which are formed during inflammatory processes, are known to be critical for signaling, aging, and apoptosis in extrinsic or intrinsic skin. Several studies showed that hydroxyl radical or endogenous ROS affected epidermal HA catabolism by producing peroxynitrite [33,34]. Overproduction of ROS/RNS has been known to promote the degradation of HA, which has the capacity to bind and retain water molecules, resulting in the expansion of skin aging. Thus, the control of ROS/RNS in skin metabolism is essential in skin health. Furthermore, HA is a major component of the extracellular matrix and is involved in water retention, maintenance of intercellular spacing, and storage and diffusion of cell growth factors and nutrients [35]. HA content decreases with skin aging and represent a direct cause of decreases in skin elasticity and moisture content [36]. HA is synthesized by HAS and degraded by HYAL [37]. Among HAS, HAS2 and HAS3 are known to play a decisive role in HA synthesis. UV rays affect the expression of these proteins, damaging the epidermal layer and causing a decrease in skin moisture content. This eventually leads to dry skin and accelerated aging [38]. However, oral milk phospholipid administration seemed to contribute to skin hydration by maintaining HA content, which was reduced by UV irradiation, as milk phospholipids upregulated HAS gene expression and downregulated HYAL gene expression (Figure 2). 

In addition, it has been reported that accumulated UV irradiation breaks the antioxidant defense system and promotes the generation of lipid oxidation products including malondialdehyde by increasing ROS production [39]. ROS generated by UVB exposure accelerate skin aging by participating in wrinkle formation and melanin generation via decomposition of binding tissue components, such as collagen and HA, and abnormal crosslinking of these components [40,41]. In this study, SOD, CAT, and GPx1 expression were decreased by UV irradiation, but milk phospholipid administration reversed this effect (Figure 4). Moreover, although sphingomyelin has been known to represent one of the main factors behind the antioxidant activity of milk and dairy products [42], the current study has demonstrated that sphingomyelin-containing milk phospholipids can upregulate the expression of antioxidant enzymes in skin tissues.

The skin barrier improvement effect of milk phospholipids appeared to be related to the activation of Nrf2-keap1, which is related to ROS removal. Milk phospholipids activated Nrf2 and increased the expression of HO-1, an antioxidant enzyme, thereby reducing ROS produced by UV irradiation (Figure 4A and Figure 5). Nrf2, which responds sensitively to intracellular oxidative stress, is a transcription factor for some antioxidant enzymes and is known to play an important role in protecting against UV-induced skin cell death and acute skin burns [43]. In a steady state, Nrf2 levels in the cytoplasm are kept low by Keap1, which forms a complex with Nrf2 and degrades it. During oxidative stress, Nrf2 is separated from Keap1 and translocated to the nucleus [44], where it forms a dimer with the small Maf protein, binds to the antioxidant response element (ARE), and activates HO-1, an ARE-dependent antioxidant gene [45]. HO-1 is a member of the intracellular phase II enzyme family, plays an important role in ROS generation and maintenance of homeostasis against oxidative stress, and is one of the cell protection mechanisms. 

We demonstrated that milk phospholipid administration improved skin hydration in a UVB-induced photoaging model. In the future, we will investigate changes in the lipid composition of the skin by administration of milk phospholipids. However, since this study evaluated the effect of milk phospholipids in a UVB-induced photoaging model, additional studies are needed to clarify the effect of milk phospholipid administration on the skin in a normal skin model. Milk phospholipids were involved in the restoration of skin barrier function damaged by UV rays and improved the skin moisture and transdermal moisture loss. This result was suspected to be because of a decrease in UV-induced ROS production following the activation of the Nrf2-Keap1 system. In addition, milk phospholipids may have improved UVB-induced skin barrier damage by supplying the skin constituent lipids containing ceramide. 

## 4. Materials and Methods

### 4.1. Materials and Animals

Sphingomyelin-containing milk phospholipids were provided by Solus Advanced Materials Co., Ltd. (Yongin, Korea). Sphingomyelin-containing milk phospholipids consist of: Phospholipids 25.0 ± 5.0% (phosphatidylcholine 7.5 ± 1.5%, phosphatidylethanolamine 6.5 ± 1.5%, phosphatidylserine 1.3 ± 0.7%, sphingomyelin 6.5 ± 1.0%), lactosylceramide 1.5 ± 0.5%, glucosylceramide 0.9 ± 0.6% and GD3 ganglioside 0.3 ± 0.1%. Eight-week-old SKH-1 hairless male mice (Central Lab Animal Inc., Seoul, Korea) were acclimatized for one week before being used in the experiment. They were reared in an environment maintained at a temperature of 23 ± 2 °C, humidity of 55 ± 10%, and light/dark cycles of 12 h. They were provided with solid feed and ad libitum access to drinking water. To induce photoaging, mice were irradiated with UVB; UVB irradiation dose was 1 minimal erythemal dose (MED; 75 mJ/cm^2^) in weeks 1 and 2, 2 MED in week 3, 3 MED in week 4, and 4 MED in week 5 onward, 3 times a week for a total of 8 weeks. For UV irradiation, a UV irradiator (BLX-254, Vilber Lourmat, Marne La Vallee, France) with UVB lamp (UB800, Waldman Licht Technik GmbH) was used. In addition, UV spectrum was measured using a UV light meter (UV-340, Lutron, Taipei, Taiwan) before UVB irradiation. Experimental animals were randomly divided into five groups, each group containing six mice: normal (unirradiated) group (NOR), UVB-irradiated group (UVB-C), 50 mg/kg b.w. milk phospholipid-administered group (ML), 100 mg/kg b.w. milk phospholipid-administered group (MM), and 150 mg/kg b.w. milk phospholipid-administered group (MH). Mice in the experimental groups (ML, MM, and MH) were orally administered with milk phospholipids once a day. The sample was administered intragastrically and was conducted simultaneously with UVB irradiation for a total of 8 weeks. The body weight of mice was measured once a week. After the end of the experiment period, whole blood was collected from the abdominal aorta and centrifuged (3000 rpm, 4 °C, 15 min) to separate plasma for serum biochemical analysis. The animal experiments were approved by the Korea University Institutional Animal Care and Use Committee (KUIACUC-2020-0054).

### 4.2. Measurement of Skin Parameters

To evaluate skin barrier function, skin hydration and TEWL were measured on the dorsal side of mice. Skin hydration content was measured using a Corneometer CM825 (Courage and Khazaka electronic GmbH, Cologne, Germany) and TEWL was measured using a Tewameter TM300 (Courage and Khazaka electronic GmbH) equipped with a Multi Probe Adapter MPA5 (Courage and Khazaka electronic GmbH). The erythema index of the mouse dorsal skin was determined using a Mexameter MX18 (Courage and Khazaka electronic GmbH). A caliper (Ozaki MFG Co., Ltd., Tokyo, Japan) was used to measure skin thickness, which was the thickness of the middle part after grabbing the skin of the lower part of the mouse tail and the neck by hand. Skin parameters (erythema, skin thickness, skin hydration, and TEWL) were expressed as delta values, which are differences from the initial values of the experiment.

### 4.3. Quantitative Real-Time PCR (qRT-PCR) Analysis

The qRT-PCR analysis was performed using cDNA prepared from mRNA fractions of tissue lysates as previously described [46]. Target gene expression was normalized to that of glyceraldehyde-3-phosphate dehydrogenase (GAPDH; NM_008084.3). The target gene information is as follows: HAS-1 (NM_008215.2), HAS-2 (NM_008216.3), HAS-3 (NM_008217.4), HYAL-1 (NM_008317.6), HYAL-3 (NM_178020.3), filaggrin (NM_001013804.2), involucrin (NM_008412.3), AQP3 (NM_016689.2), superoxide dismutase (SOD) 1 (NM_011434.1), SOD2 (NM_013671.3), glutathione perxodiase1 (GPx-1) (NM_008160.6), and catalase (CAT) (NM_009804.2).

### 4.4. Measurement of Protein Expression by Western Blot Analysis

Proteins were isolated from skin tissues using a lysis buffer, and the concentration of the isolated proteins was quantified using the Bradford assay [47]. Proteins were separated by 6–15% sodium dodecyl sulfate-polyacrylamide gel electrophoresis and then transferred to polyvinylidene fluoride membranes. After blocking the membranes with 5% skim milk solution for 1 h, anti-Nrf2 (1:1000, #12721, Cell Signaling Technology, Beverly, MA, USA), anti-Keap1 (1:1000, #8047, Cell Signaling Technology), anti-HO-1 (1:1000, SC-120745, Santa Cruz Biotechnology, Dallas, TX, USA), and anti-β-actin (1:1000, #8457, Cell Signaling Technology) were added, and incubated overnight at 4°C. The membranes were washed with 1X Tris-buffered saline (TBST) buffer, reacted with secondary antibodies (anti-rabbit IgG, 1:2000, #7074, Cell Signaling Technology) for 2 h, washed with 1X TBST buffer, and treated with enhanced chemiluminescence reagent to determine the expression of proteins. The results were normalized to the endogenous protein, β-actin.

### 4.5. Measurement of ROS

For ROS measurement, the skin tissues were homogenized in 40 mM Tris-HCl buffer (pH 7.4) and centrifuged [48]. Next, 10 μM 2′,7′-dichlorodihydrofluorescein diacetate (Sigma-Aldrich, St Louis, MO, USA) was added to the supernatant and reacted at 37 °C. Fluorescence (Excitation wavelength: 485 nm, emission wavelength: 535 nm) was measured after 30 min (SpectraMax Gemini EM fluorometer, Molecular Devices, Sunnyvale, CA, USA).

### 4.6. Statistical Analysis

Data are expressed as the mean ± standard mean error (SEM). The statistical significance was at the *p* < 0.05 level. Comparisons between treatment groups were performed using one-way ANOVA followed by the Tukey’s multiple range test using the Statistical Package for the Social Science software (SPSS Version 20, SPSS Inc., Chicago, IL, USA).

## Data Availability

The data that support the findings of this study are available from the corresponding author upon reasonable request.

## Supplementary Materials

The following supporting information can be downloaded at: https://www.mdpi.com/article/10.3390/molecules27082545/s1, Table S1. Effects of milk phospholipids on body weight changes in ultraviolet (UV) B-irradiated hairless mice; Table S2. Effects of milk phospholipids on serum biochemical parameters in ultraviolet (UV) B-irradiated hairless mice.

## Sample Availability

Samples of the milk phospholipids are available from the corresponding authors, Ki-Bae Hong and Hyung Joo Suh.
