# Supplementary material for: Effects of Sphingomyelin-Containing Milk Phospholipids on Skin Hydration in UVB-Exposed Hairless Mice
Source: Molecules. 2022 Apr 14;27(8):2545. doi: 10.3390/molecules27082545 (PMC9032803; doi:10.3390/molecules27082545)
Supplement: Supplementary file 1 [file molecules-27-02545-s001.zip › molecules-1679332-supplementary.pdf]

Supplementary Materials

Effects of Sphingomyelin-Containing Milk Phospholipids on Skin Hydration in UVB-Exposed Hairless Mice

Yejin Ahn [1], Min Guk Kim [1], Kyungae Jo [1], Ki-Bae Hong [2,*] and Hyung Joo Suh [1,3,*]

[1] Department of Integrated Biomedical and Life Science, Graduate School, Korea University, Seoul 02841, Korea; ahnyj708@gmail.com (Y.A.); minguk94@gmail.com (M.G.K.); kyungae11@korea.ac.kr (K.J.)

[2] Department of Food Science and Nutrition, Jeju National University, Jeju 63243, Korea

[3] BK21FOUR R&E Center for Learning Health Systems, Korea University, Seoul 02841, Korea

* Correspondence: kbhong@jejunu.ac.kr (K.-B.H.); suh1960@korea.ac.kr (H.J.S.); Tel.: +82-23-290-5639 (H.J.S.)

Table S1. Effects of milk phospholipids on body weight changes in ultraviolet (UV) B-irradiated hairless mice.

Weeks	NOR	UVB-C	ML	MM	MH
1	31.63±0.48 ^{ns}	31.43±0.50	31.75±0.82	31.90±0.31	31.08±0.56
2	31.83±0.78 ^{ns}	31.48±0.11	32.48±0.77	31.98±0.75	31.73±0.57
3	32.93±0.49 ^{ns}	32.03±0.42	33.28±0.91	32.68±0.85	32.83±0.91
4	33.63±0.36 ^{ns}	33.10±0.58	33.55±1.10	33.30±0.79	32.63±0.82
5	34.43±0.51 ^{ns}	33.75±0.48	34.33±0.98	34.85±0.75	33.63±0.86
6	35.60±0.45 ^{ns}	34.58±0.73	34.50±0.91	34.90±0.60	34.35±0.86
7	36.05±0.77 ^{ns}	35.83±0.63	35.88±0.67	35.70±0.71	34.95±0.93
8	36.13±0.62 ^{ns}	34.75±0.57	35.78±1.22	36.28±0.66	34.55±0.42

NOR: oral administration of saline without UVB irradiation; UVB-C: oral administration of saline under UVB irradiation; ML: oral administration of low-dose (50 mg/kg b.w.) milk phospholipids under UVB irradiation; MM: oral administration of medium-dose (100 mg/kg b.w.) milk phospholipids under UVB irradiation; MH: oral administration of high-dose (150 mg/kg b.w.) milk phospholipids under UVB irradiation. Data are expressed as means ± standard error (n=6). ns, not significant.

Table S2. Effects of milk phospholipids on serum biochemical parameters in ultraviolet (UV) B-irradiated hairless mice.

Groups	GLU (mg/dL)	TG (mg/dL)	TCHO (mg/dL)	AST (U/L)	ALT (U/L)
NOR	429.00±34.22 ns	245.50±11.87	136.75±6.55 ns	39.50±3.50 ns	57.75±2.17 ns
UVB-C	442.00±2.16	188.25±18.15	143.00±8.19	45.50±1.71	59.25±2.06
ML	431.00±23.19	200.50±24.74	130.50±4.43	45.50±2.36	60.25±2.78
MM	494.00±37.53	154.50±18.04#	129.00±1.73	41.00±1.29	57.75±3.42
MH	429.00±41.19	132.60±13.30##	126.50±6.34	47.00±5.00	64.50±1.71

NOR: oral administration of saline without UVB irradiation; UVB-C: oral administration of saline under UVB irradiation; ML: oral administration of low-dose (50 mg/kg b.w.) milk phospholipids under UVB irradiation; MM: oral administration of medium-dose (100 mg/kg b.w.) milk phospholipids under UVB irradiation; MH: oral administration of high-dose (150 mg/kg b.w.) milk phospholipids under UVB irradiation. Data are expressed as means ± standard error (n=6). #p<0.05 and ##p<0.01 vs. NOR group (Tukey's test). GLU, glucose; TG, triglyceride; TCHO, total cholesterol; AST, aspartate transaminase; ALT, alanine transferase; ns, not significant.
